# Postpartum Left Ventricular Dysfunction in Preeclampsia With Incidental Patent Ductus Arteriosus: A Multimodality Hemodynamic Assessment

**DOI:** 10.7759/cureus.105625

**Published:** 2026-03-21

**Authors:** Giovanni Paolella, Viktoriya Bikeyeva, Andrii Labchuk, Katarzyna Mikrut

**Affiliations:** 1 Internal Medicine, Advocate Lutheran General Hospital, Park Ridge, USA; 2 Cardiology, Advocate Lutheran General Hospital, Park Ridge, USA

**Keywords:** cardiac magnetic resonance imaging, congenital heart disease, patent ductus arteriosus, postpartum cardiomyopathy, preeclampsia

## Abstract

Hypertensive disorders of pregnancy, particularly preeclampsia, are associated with increased risk of postpartum cardiovascular complications. We report a case of a 43-year-old female with known mild-to-moderate aortic regurgitation and thoracic aortic dilation who developed new left ventricular systolic dysfunction shortly after cesarean delivery for severe preeclampsia. On postoperative day three, she presented with chest pain and was found to have a reduced left ventricular ejection fraction (43%) on transthoracic echocardiography. Computed tomography angiography excluded aortic dissection but revealed a patent ductus arteriosus not previously described on earlier imaging, raising concern for possible shunt-mediated volume overload. Cardiac magnetic resonance imaging performed four months postpartum demonstrated low-normal systolic function with Qp/Qs of 0.76, and right heart catheterization at seven months confirmed Qp/Qs of 1 with normal pulmonary vascular resistance, indicating no hemodynamically significant shunt. The patient was managed with guideline-directed medical therapy for heart failure and remained clinically stable with recovery of ventricular function. This case highlights the importance of multimodality imaging and invasive hemodynamic assessment before pursuing structural intervention in postpartum patients with newly identified cardiac abnormalities.

## Introduction

Hypertensive disorders of pregnancy, particularly preeclampsia, are increasingly recognized as important contributors to both immediate and long-term cardiovascular morbidity. Women with a history of preeclampsia face elevated risks of postpartum heart failure, left ventricular (LV) dysfunction, chronic hypertension, ischemic heart disease, and stroke [1,2]. The early postpartum period is characterized by substantial hemodynamic changes, including mobilization of extracellular fluid, autotransfusion from uterine involution, and dynamic shifts in systemic vascular resistance [3]. These physiologic changes can precipitate cardiac decompensation even in individuals without prior structural disease.

In patients with known structural abnormalities, including valvular disease or congenital heart lesions, postpartum hemodynamic stress may unmask latent dysfunction. Patent ductus arteriosus (PDA) is a congenital connection between the descending aorta and pulmonary artery that typically closes shortly after birth. When persistent and hemodynamically significant, it may result in LV volume overload and progressive dilation. However, closure is recommended only when objective hemodynamic criteria are met, including significant left-to-right shunt (Qp/Qs >1.5), LV enlargement, and acceptable pulmonary vascular resistance [4,5].

The coexistence of preeclampsia, structural valvular disease, and an incidental PDA presents a diagnostic challenge. Distinguishing transient postpartum myocardial dysfunction from shunt-mediated volume overload is essential to guide appropriate management. We present a case highlighting the importance of multimodality imaging and invasive hemodynamic confirmation before structural intervention.

## Case presentation

**Figure 1:**
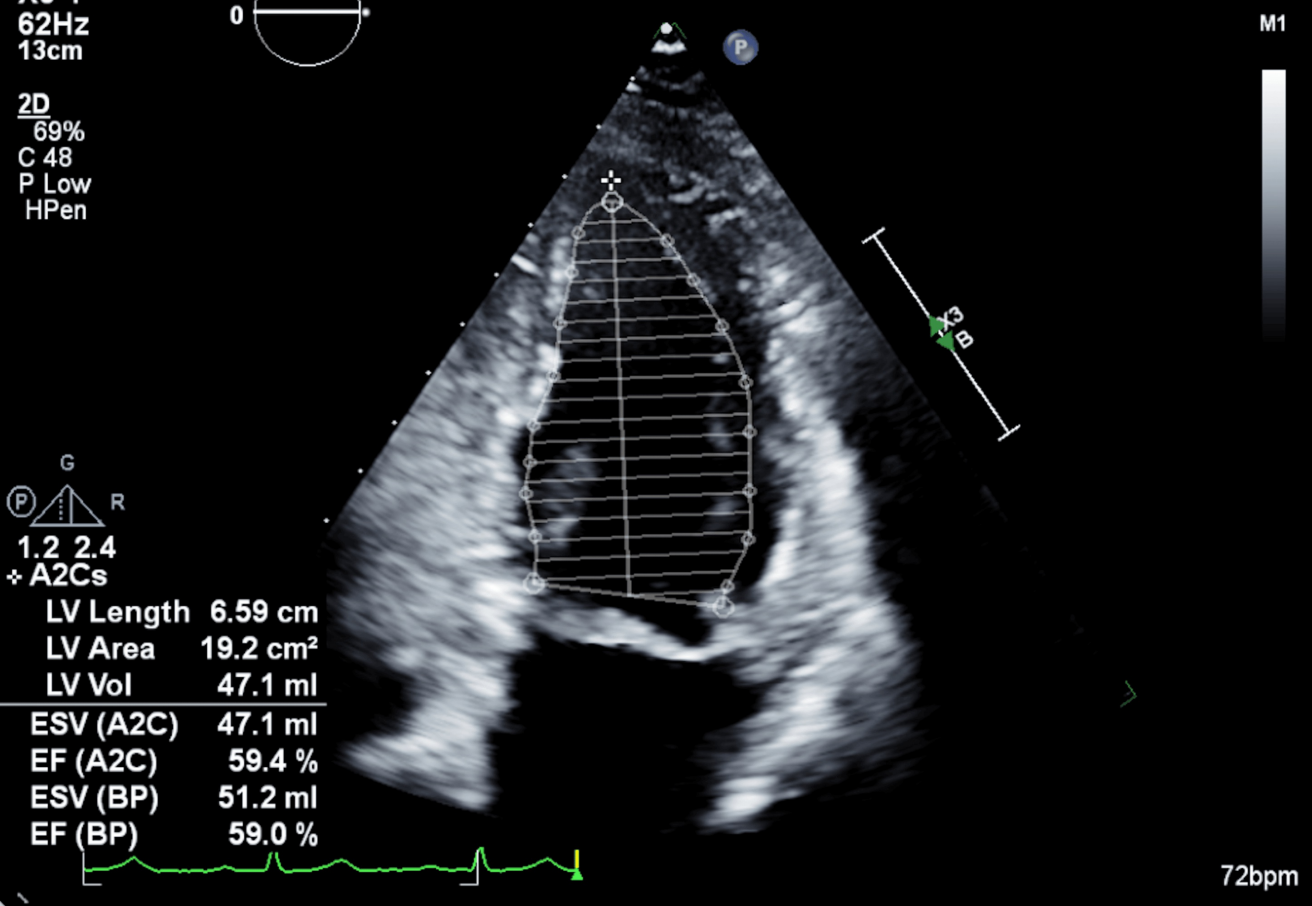
Pre-hospital TTE (A2C view) with endocardial tracing using Simpson’s method showing preserved LV systolic function. The LVEF is preserved at approximately 59% (EF (BP) 59.0% and EF (A2C) 59.4% as shown), with an ESV reported on-screen (ESV (A2C) 47.1 mL; ESV (BP) 51.2 mL). TTE: transthoracic echocardiography; ESV: end-systolic volume; BP: blood pressure; LVEF: left ventricular ejection fraction; EF: ejection fraction; A2C: apical two-chamber

A 43-year-old female patient (gravida 5 para 2) with a history of nonrheumatic aortic valve insufficiency, mildly dilated thoracic aorta (4.1 cm), prior deep vein thrombosis, and seizure disorder presented at 36 weeks' gestation with severe preeclampsia. Earlier in pregnancy, transthoracic echocardiography (TTE) demonstrated preserved LV systolic function (LV ejection fraction (LVEF) 59%) and mild-to-moderate aortic regurgitation (Figure 1).

**Figure 2:**
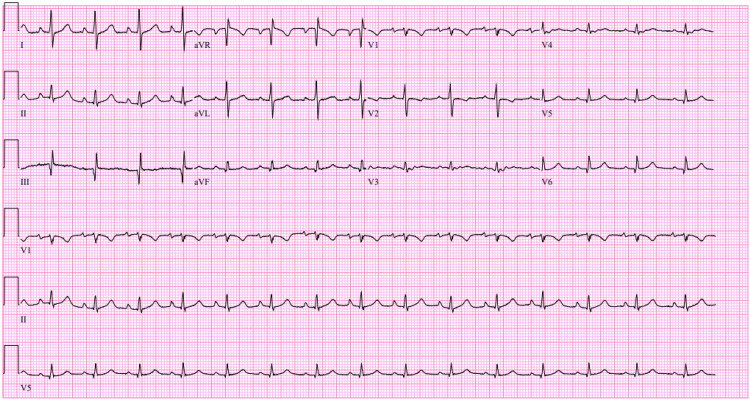
Electrocardiogram (12-lead) in postpartum chest pain demonstrating normal sinus rhythm with a ventricular rate in the normal range and narrow QRS complexes There are nonspecific ST-T wave abnormalities without ST-segment elevation, depression consistent with acute ischemia. These findings supported further evaluation for non-ischemic causes of newly reduced left ventricular systolic function in the early postpartum period.

The patient underwent cesarean delivery without immediate complication. On postoperative day three, she developed new-onset chest pain radiating to her back and trace lower extremity edema. Blood pressure was mildly elevated but hemodynamically stable. N-terminal pro-B-type natriuretic peptide (NT-proBNP) was 68 pg/mL (reference range <125 pg/mL), and troponin was <4 ng/L (reference range <52 ng/L), both within normal limits. The electrocardiogram showed no ischemic changes (Figure 2). Her initial blood tests and electrocardiogram made acute coronary syndrome less likely.

**Figure 3:**
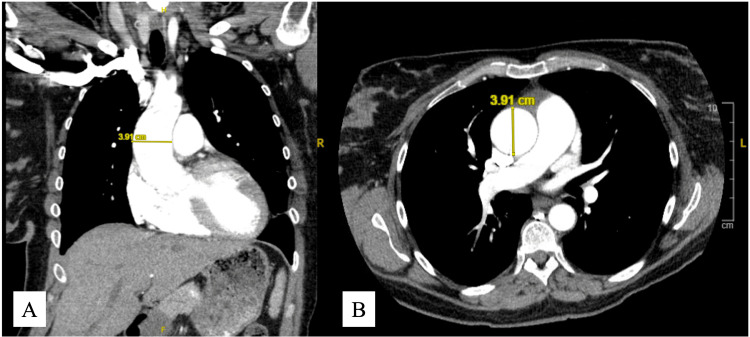
CECT of the thoracic aorta demonstrating mild dilation of the ascending aorta measuring 3.91 cm (A) Coronal view; (B) Axial view. The aortic contour is smooth without evidence of dissection, intramural hematoma, or penetrating ulcer. These images were obtained during evaluation of postpartum chest pain in a patient with known aortic valve insufficiency and newly reduced left ventricular systolic function. CECT: contrast-enhanced computed tomography

Given concern for acute aortic pathology in the setting of known aortic dilation, computed tomography angiography (CTA) was performed (Figure 3).

**Figure 4:**
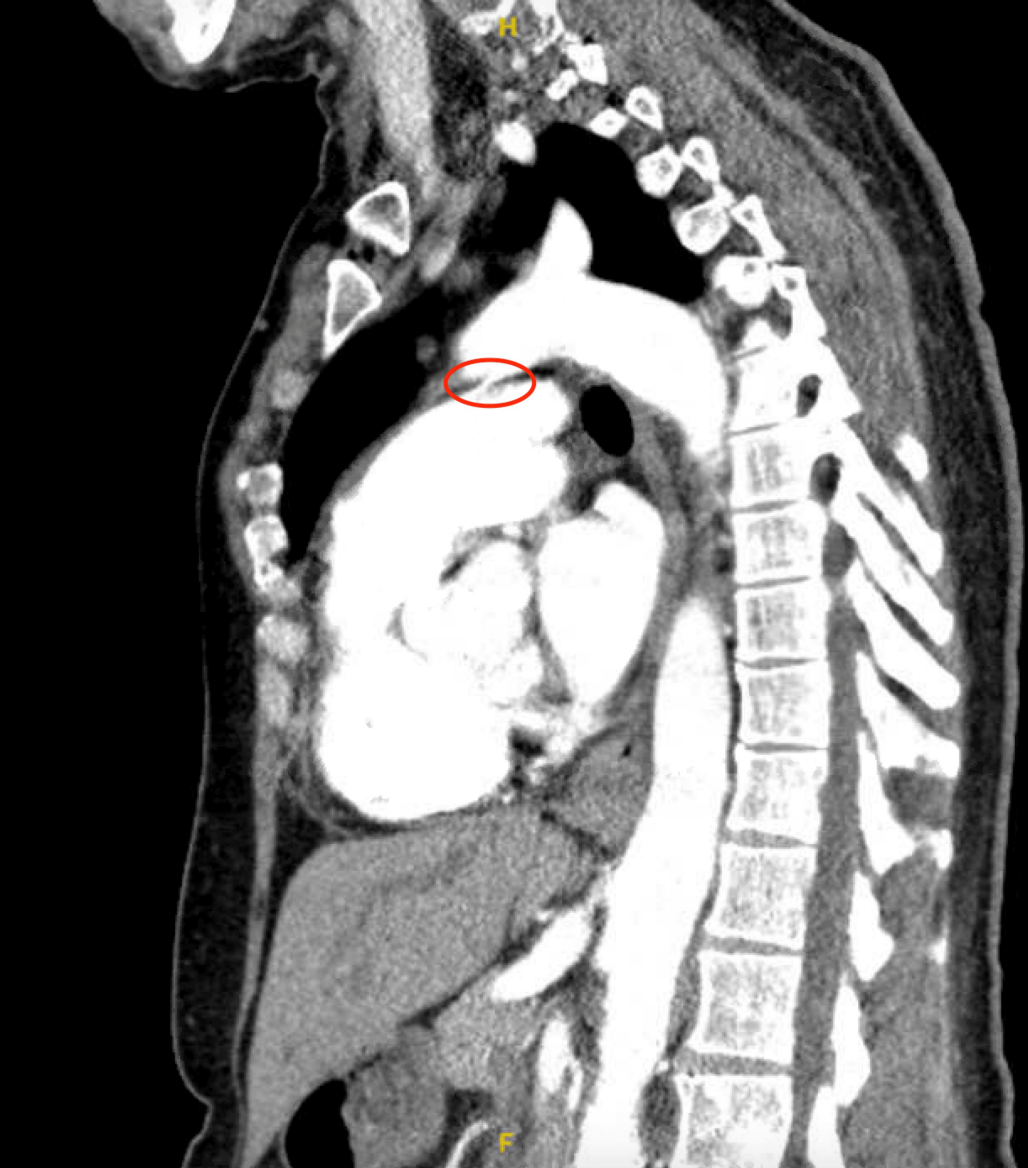
Contrast-enhanced CTA of the thoracic aorta (sagittal view) demonstrating a small PDA (red circle) in the early postpartum period The PDA is seen arising distal to the left subclavian artery and connecting to the proximal pulmonary artery. The ascending aorta, aortic arch, and descending thoracic aorta are visualized without evidence of dissection. This study was obtained during evaluation of postpartum chest pain and newly reduced left ventricular ejection fraction. CTA: computed tomography angiography; PDA: patent ductus arteriosus

CTA excluded aortic dissection and demonstrated stable aortic dimensions; however, it revealed a PDA not previously described on prior imaging (Figure 4). The finding raised concern regarding possible left-to-right shunting contributing to volume overload.

**Figure 5:**
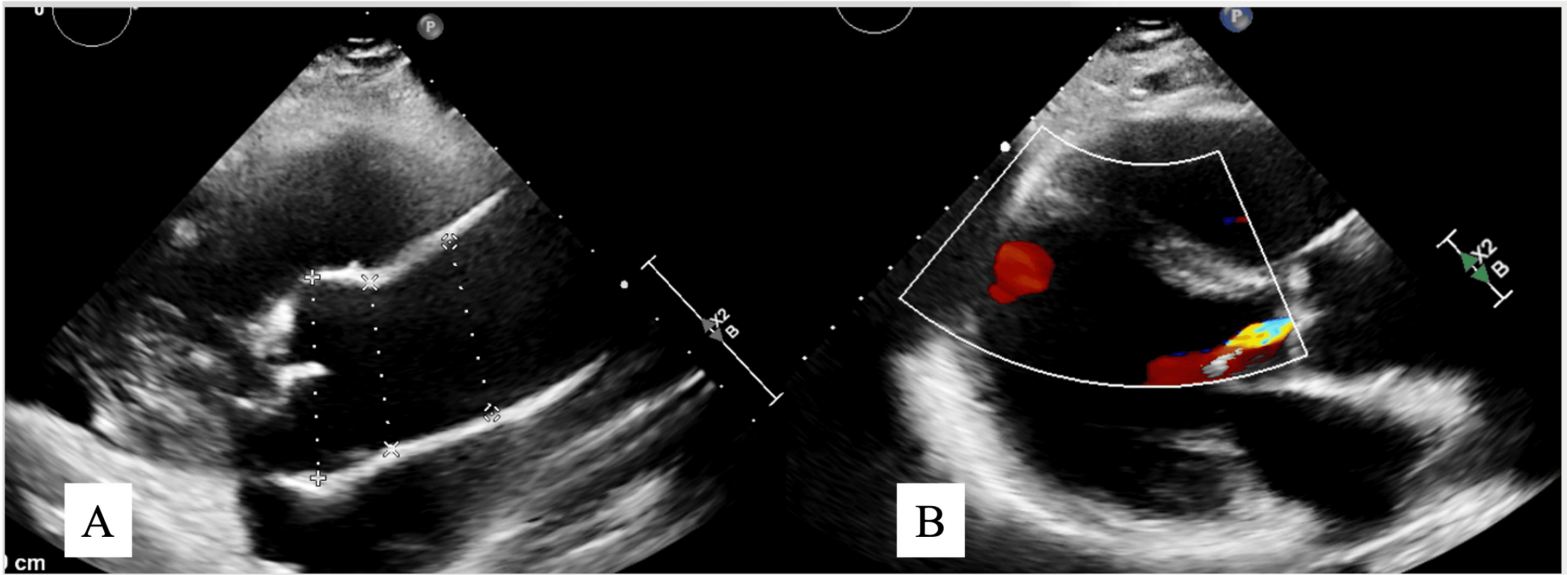
TTE demonstrating left ventricular dysfunction and aortic regurgitation Parasternal long-axis TTE images with two-dimensional measurements (A) color Doppler (B) and demonstrating reduced left ventricular systolic function (LVEF 43%) and moderate-to-severe aortic regurgitation. The left ventricular cavity size is at the upper limits of normal, and the aortic root is mildly dilated (3.9 cm). Mild mitral regurgitation is present, with normal right ventricular size and systolic function and mild pulmonic insufficiency. TTE: transthoracic echocardiography; LVEF: left ventricular ejection fraction

Repeat TTE demonstrated newly reduced LV systolic function with LVEF 43%, moderate-to-severe aortic regurgitation, and mild aortic root dilation (Figure 5). The decline in LVEF from 59% earlier in pregnancy to 43% postpartum suggested new systolic dysfunction. The temporal association between preeclampsia, postpartum physiology, and the newly described PDA created diagnostic uncertainty regarding causation.

**Figure 6:**
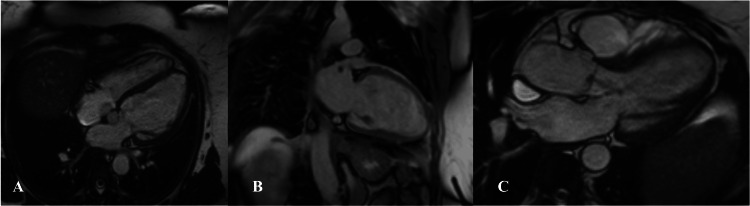
CMR demonstrating borderline left ventricular function, ventricular dilatation, and aortic regurgitation (A) Axial cine CMR demonstrating dilated ventricles with preserved myocardial contours, consistent with borderline left ventricular systolic function. (B) Sagittal cine CMR view showing left ventricular chamber enlargement, supporting the presence of ventricular dilatation associated with borderline ejection fraction. (C) Cine CMR demonstrating a visible aortic regurgitation jet during diastole, indicating the presence of aortic regurgitation without quantitative assessment. CMR: cardiac magnetic resonance imaging

To further evaluate ventricular volumes, regurgitant fraction, and potential shunt physiology, cardiac magnetic resonance imaging (CMR) was performed four months postpartum. CMR demonstrated mildly to moderately dilated LV size with low-normal systolic function (LVEF 54%). Mild-to-moderate aortic regurgitation was quantified with a regurgitant fraction of 11%. The right ventricle was mildly dilated with preserved systolic function (right ventricular ejection fraction (RVEF) 51%) (Figure 6).

The ascending aorta measured 4.1 × 3.8 cm. Phase-contrast and cine imaging suggested a PDA with Qp/Qs 0.76, a value below the threshold for hemodynamically significant left-to-right shunting. Although Qp/Qs <1 does not indicate a significant left-to-right shunt, variability in CMR flow measurements for small shunts is recognized [6].

To definitively assess hemodynamic significance, right heart catheterization was performed seven months postpartum. Hemodynamics revealed Qp/Qs of 1 and pulmonary vascular resistance <3 Wood units, confirming the absence of a significant shunt. A very small PDA ostium was visualized but could not be engaged. Given these results, the PDA was deemed incidental and not responsible for LV dysfunction. The patient was transitioned to full guideline-directed medical therapy (GDMT) in accordance with contemporary heart failure guidelines [7,8]. She remained clinically stable with preserved LV function on follow-up.

## Discussion

This case underscores the interplay between postpartum physiology, hypertensive disorders of pregnancy, and structural heart disease. The early postpartum period is associated with abrupt hemodynamic shifts, including increased preload and afterload, which may precipitate myocardial dysfunction [3]. In women with preeclampsia, endothelial dysfunction and elevated afterload further increase myocardial stress [1,2]. The decline in LVEF observed in this patient likely reflects transient myocardial dysfunction related to postpartum hemodynamic stress, potentially overlapping with mechanisms seen in peripartum cardiomyopathy [6]. However, distinguishing primary myocardial dysfunction from structural volume overload is essential when a shunt lesion is identified.

In this patient, the development of LV dysfunction in the postpartum period may reflect transient myocardial impairment related to these hemodynamic changes, with overlap in mechanisms described in peripartum cardiomyopathy [7]. However, the incidental identification of a PDA introduced diagnostic uncertainty, as significant left-to-right shunting can also lead to volume overload and ventricular dilation. The Qp/Qs ratio provides a key measure of shunt severity, with values greater than 1.5 generally indicating hemodynamically significant shunting requiring intervention [4]. In this case, CMR demonstrated a Qp/Qs of 0.76, suggesting no significant shunt. However, given known variability in CMR flow quantification, particularly in small shunts, definitive assessment requires invasive hemodynamic evaluation [6]. Right heart catheterization confirmed a Qp/Qs of 1 with normal pulmonary vascular resistance, definitively excluding a hemodynamically significant PDA. This distinction was critical, as it prevented unnecessary structural intervention. 

Importantly, each diagnostic modality contributed uniquely: echocardiography identified systolic dysfunction, CTA identified structural anatomy and excluded dissection, CMR quantified ventricular function and regurgitation, and catheterization confirmed hemodynamic significance. This stepwise integration of data illustrates the value of multimodality assessment in complex postpartum presentations. 

Management appropriately focused on GDMT for heart failure. The 2021 European Society of Cardiology (ESC) heart failure guidelines recommend early initiation of quadruple therapy for patients with reduced LVEF unless contraindicated [8]. The patient’s improvement in LV function supports the effectiveness of medical therapy and reinforces that incidental structural findings should not distract from evidence-based heart failure management.

## Conclusions

Postpartum LV dysfunction in patients with preeclampsia may mimic or coexist with structural abnormalities, creating diagnostic uncertainty. Multimodality imaging combined with invasive hemodynamic assessment is essential to distinguish transient myocardial dysfunction from clinically significant shunt physiology. Consideration of alternative diagnoses, including peripartum cardiomyopathy, is important in this setting. Careful integration of diagnostic data can prevent unnecessary interventions while ensuring appropriate medical management. 
